# Effect of sodium stibogluconate in recruiting and awakening immune cells in the pleural fluid of pancreatic cancer: preparation for immunotherapy

**DOI:** 10.3389/fimmu.2023.1315468

**Published:** 2024-01-19

**Authors:** Baofa Yu, Peng Jing, Feng Gao, Peicheng Zhang, Guoqin Zheng, Xiaomin Zhang

**Affiliations:** ^1^ Department of Oncology, TaiMei Baofa Cancer Hospital, Dongping, Shandong, China; ^2^ Department of Oncology, Jinan Baofa Cancer Hospital, Jinan, Shandong, China; ^3^ Department of Oncology, Beijing Baofa Cancer Hospital, Beijing, China; ^4^ Department of Internal Medicine, South China Hospital of Shenzhen University, Shenzhen, China; ^5^ Immune Oncology Systems, Inc., San Diego, CA, United States

**Keywords:** sodium stibogluconate, immunotherapy with sodium stibogluconate, protein tyrosine phosphatase inhibitor, immune inducer, immune booster

## Abstract

Ascites and pleural effusion are recognized complications of pancreatic cancer. These diseases are accompanied by ascites and pleural effusion, and drug treatment is limited by high costs, long hospital stays, and failure rates. Immunotherapy may offer new option, but in most patients with late stages of cancer, immune cells may lose the ability to recognize tumor cells, how to activate their immune cells is a major problem, sodium glucosidate (SSG) is injected into ascites as a protein tyrosine phosphatase inhibitor to wake up immune cells and prepare for immunotherapy. We used single-cell RNA sequencing (scRNA-seq) to investigate whether and how SSG injected into ascites of pancreatic cancer elicits an immune response. Our study showed that the process of SSG fusion treatment of ascites and pleural effusion, the interaction between TandNK cells, MPs cells, monocytes and neutrophils was induced, and large numbers of genes were expressed, resulting in upregulation of immune response, which also approved that SSG is not only used as a protein tyrosine phosphatase inhibitor, but also it works as a protein tyrosine phosphatase inhibitor. It can also be used to regulate immune cell function, recruiting immune cells to the right place with the help of PD-1 or PD-L1 to fight cancer cells in ascites and pleural effusions in cancer patients.

## Introduction

The incidence and death rates for pancreatic cancer have been gradually increasing while the incidence and death rates for other common cancers have been declining. Only about 4% of pancreatic cancer patients live five years after diagnosis. For some patients, malignancies are only located to the pancreas without metastasis and their survival rates are higher due to surgical removal. Unfortunately, 80-85% of patients have advanced, unresectable cancer. In addition, pancreatic cancer does not respond well to most chemotherapy drugs (1). We have recently experimented the injection of compound chemotherapy drugs plus hapten into advanced pancreatic cancer, which killed new tumors and induced an immune response, thereby awakening immune cells and prolonging the life of patients (2). For advanced pancreatic cancer patients with pleural metastasis and pleural effusion, there are no good therapies and drugs to choose from, even the advanced immunotherapy will not be helpful (3).

Ascites and pleural effusion are recognized as a severe complication of pancreatic disease. Drug treatment for these conditions is limited by high costs, long hospital stays and failure rates; Invasive procedure is accompanied with a high morbidity and mortality rate. Endoscopic therapy is often the first-line treatment of pancreatic ascites and pleural effusion. The intervention is a 5 mm pancreatic sphincterotomy and endoscopic placement of a 7 Fr pancreatic stent, through which bridges the pancreatic ductal leak. The method offers a higher rate of success and provides a good treatment option for patients with pancreatic ascites and pleural effusion (3, 4).

However, it is still a form of physical therapy and can only provide relief. Thoracic infusion of chemotherapy drugs is also commonly used for the treatment of pancreatic cancer patients with ascites and pleural effusion, but the side effects are more severe that renders the method less effective, thus limiting its use. This paper will discuss the application of non-chemotherapy drug perfusion in pleural effusion, recruit and wake up the aggregation of immune cells, and use immunotherapy to provide an opportunity for the immunotherapy of pancreatic ascites and pleural effusion.

In this study, we aimed to determine whether sodium stibogluconate (SSG) was used for pleural effusion infusion to effectively control pancreatic cancer ascites and pleural effusion and awake up various immune cells and recruit them to pleural effusion for therapeutic effect. SSG was commonly used for treatment of visceral leishmaniasis in a population with a high prevalence of HIV infection in Ethiopia and intralesional sodium stibogluconate for the treatment of localized cutaneous leishmaniasis at Boru Meda general hospital (5, 6). SSG was injected with single drug into pleural effusion, pleural fluid samples were taken before and after treatment of SSG injection into pleural fluid. Then we used single-cell RNA sequencing (scRNA-Seq) to obtain transcriptome profiles of a total of 42249 EpithelialCells, and the proportion of changes in immune cells before and after treatment. Through comparative analysis of different samples of CIN and ascite samples, we comprehensively described the expression characteristics of malignant epithelial cells and immune cells, including Epithelial Cells, Ecs, Fibroblasts, Mural Cells, Tcells, Bcells, TandNK Neutrophils, Mast Cells, MPs, and Platelets, as well as the dynamic changes in cell percentage and cell subtype heterogeneity. Our results provide evidence that pleural effusion infusion was treated with single drug of SSG for induce acute immune response in pancreatic cancer ascites and pleural effusion, recruit and awaken immune cell for fighting cancer cells in the pleural fluid of pancreatic cancer.

## Materials and methods

### Ethical statement

All procedures and protocols in the study have been reviewed and approved by the Ethical Committee of the Shandong Baofa Cancer Institute (TMBF 0010, 2021). All informed consent forms from patients have been signed prior to the start of the study.

### Clinical specimens

Pancreatic head cancer with retroperitoneal lymph node metastasis and liver metastasis. The tumor marker CEA was significantly elevated, the vital signs were stable, the skin and mucosa of the whole body had no yellow stain and bleeding points, the superficial lymph nodes were not swollen, the chest wall metastases were not detected, and there was a large amount of pleural fluid. It is not suitable for treatment of surgery and chemotherapy, and only palliative care can alleviate pain and prolong life. This experimental treatment was approved by the hospital ethics committee (TMBF 0010, 2015) in accordance with relevant guidelines and regulations.

Pleural infusion was injected with 5.7g of Sodium stibogluconate (3 sticks) once, once every other day, twice in total, and no adverse reactions such as fever and bleeding were observed. Ascite samples (25-30 ml) were taken before the treatment as control and after treatment as the treated samples for scRNA-Seq analysis, each samples contained over 10,000 cells. A total of three samples from the patient was taken as well at before and 4 days and 8 days after injection of Sodium stibogluconate.

### Tissue disassociation and cells collection

**Figure 1:**
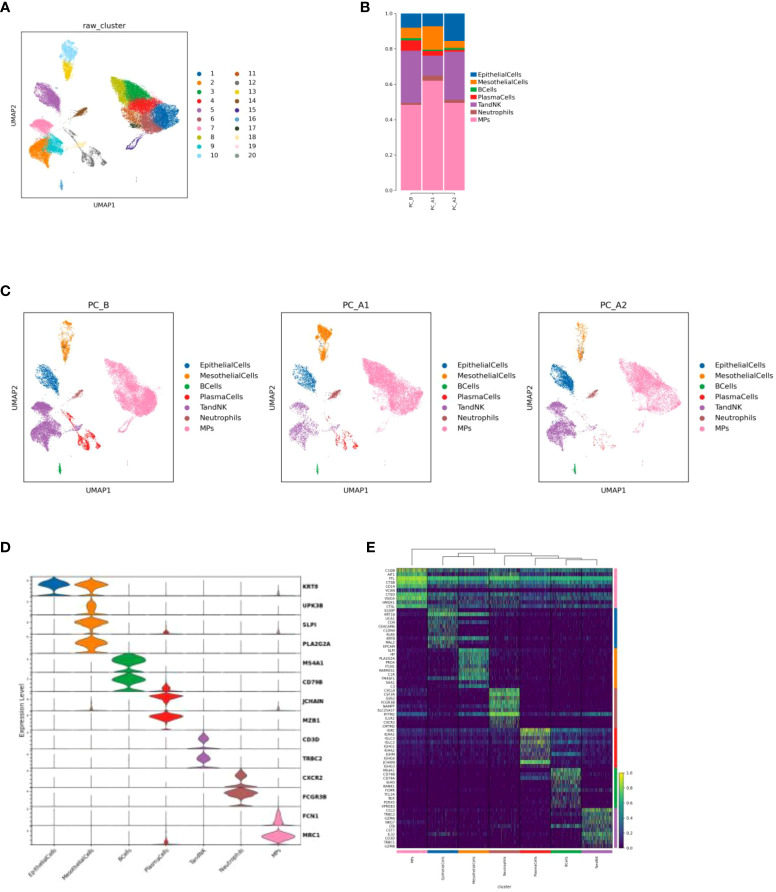
**(A)** Cell Type Reduction Map Display (UMAP). **(B)** Histogram of the proportion of cell types in different groups. **(C)** Display the dimensionality reduction map of major cell types in groups (UMAP, in the order of PC_B, PC_A1, PC_A2). **(D)** Violin map of marer genes in different cell types. **(E)** top10 heat maps of different cell types.

After sample extraction (**Figures 1C, D**), the fresh cells samples were immediately stored in the sCelLiVE® Tissue Preservation Solution (Singleron) on ice. The sample of ascites were transferred to a 15-ml centrifuge tube. The samples were then filtered with 40 µm sterile strainers, and centrifuged at 1,000 rpm at 4°C for 5 min. Next, 2 ml GEXSCOPE® red blood cell lysis buffer (RCLB, Singleron) was added to lyse the red blood cells for 10 min. Finally, the single cell suspension was collected after re-suspension with PBS, and trypan blue (Sigma) staining was used to calculate cell activity and cell count under a microscope (7).

### Single-cell RNA sequencing

Single-cell suspensions (1~3×105 cells/mL) in PBS (HyClone) were loaded onto microwell chip using the Singleron Matrix® Single Cell Processing System. Briefly, the scRNA-seq library was constructed using the GEXSCOPE® Single Cell RNA Library Kits (Singleron). The library was lastly sequenced with 150 bp that was diluted to 4nM and paired-end reads on the IlluminaHiSeq X platform following an established protocol (7). Sequencing data processing and quality control was performed as described in previous publications (8, 9).

### Data processing and analysis

To identify differentially expressed genes (DEGs), genes expressed in more than 10% of the cells were selected in both groups of cells and with an average log (fold changes) value greater than 1 as DEGs.

The cell type identity of each cluster was determined with the expression of canonical markers found in the DEGs using SynEcoSys database (Singleron Biotechnologies).

The InferCNV package was used to detect the CNAs in malignant cells. Non-malignant cells (T and NK cells) were used as control references to estimate the CNVs of malignant cells. Genes expressed in more than 20 cells were sorted based on their loci on each chromosome. The relative expression values were centered to 1, using 1.5 standard deviation from the residual-normalized expression values as the ceiling.

To investigate the potential functions of DEGs between clusters, the Gene Ontology (GO) and Kyoto Encyclopedia of Genes and Genomes (KEGG) analysis were used with the “clusterProfiler” R package 3.16.1 (10).

Monocle 2 algorithm was used for pseudo-time trajectory analysis, and the dimensionality reduction method used was DDRTree (11).

Intra-tumoral heterogeneity (ITH) score calculation: The ITH score was defined as the average Euclidean distance between the individual cells and all other cells, in terms of the first 20 principal components derived from the normalized expression levels of highly variable genes.

Cell-cell interaction (CCI) between B cells, Epithelial cells, Fibroblasts, Mononuclear phagocytes, Mast cells, Neutrophils, T and NK cells were predicted based on known ligand–receptor pairs by Cellphone DB v2.1.0 (12).

## Results

### Clinical benefit

After SSG treatment, patient felt a relive from in-comfortable condition, ascites and pleural effusion in the patient is stable and can last for a long time without pumping, no more side effect like chemotherapy.

### Landscape of single cell transcriptome sequencing before and after treatment

There were 42,249 cells in 3 samples after quality control filtration. The cells were divided into 20 clusters after unsupervised clustering and dimensionality reduction. 7 cell types were identified based on marker gene expression results. Including epithelial cells (KRT8, UPK3B), mesothelial cells (SLPI, PLA2G2A), B cells (MS4A1, CD79B), plasma cells (JCHAIN, MZB1), TandNK cells (CD3D, TRBC2), neutrophils (CXCR2, FCGR3B) and mononuclear phagocytic cells (FCN1, MRC1) (**Figures 1A, C, D, E**). It was found that with the course of treatment, the proportion of epithelial cells and TandNK cells first decreased and then increased, the proportion of neutrophils and mononuclear phagocytes first increased and then decreased (**Figure 1B**), and the proportion of plasma cells decreased.

### Changes of epithelial cells before and after treatment

**Figure 2:**
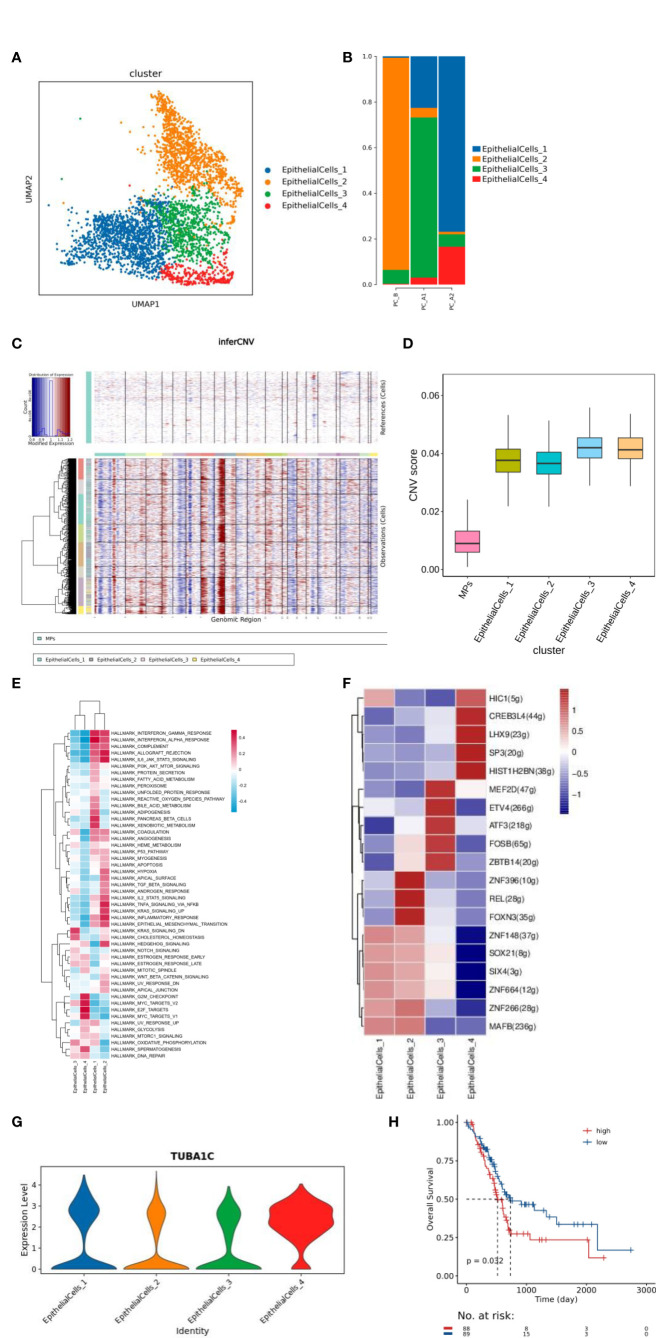
**(A)** Dimensionality reduction map of epithelial cell subsets. **(B)** Histogram of proportion of epithelial cell subtypes. **(C)** inferCNV and CNV score results of epithelial cells. **(D)** ITH results of epithelial cell grouping. **(E)** Epithelial cell subtype GSVA analysis (hallmark). **(F)** Transcription factor analysis results of epithelial cell subsets. **(G)** Violin map of TUBA1C gene expression in epithelial cell subtype, **(H)** Prognostic analysis of h. TUBA1C gene TCGA.

The total number of EpithelialCells is 3903, and there are 4 different subtypes, including EpithelialCells_1-4 (**Figure 2A**). Through cell proportion, CNV and ITH analysis, it was found that pleuroepithelial cells of patients with different treatment processes showed obvious heterogeneity, among which subgroup 2 was mainly in PC_B samples, subgroup 3 was mainly in PC_A1 samples, and subgroups 1 and 4 were mainly in PC_A2 samples (**Figures 2B–D**).

The function of each cluster was identified based on GSVA analysis (hallmark database) (13), and subgroup 1 was found to be enriched in IFN-α and IFN-γ signaling pathways, subgroup 2 was enriched in EMT signaling and IL6/JAK2/STAT3 signaling pathways, and subgroup 3 was enriched in cholesterol balance and oxidative phosphorylation pathways. Subgroup 4 was enriched in MYC signaling pathway and G2M signaling pathway (**Figure 2E**). 4 clusters raised CREB3L4 activity of transcription factors, CREB3L4 TUBA1C genes (**Figures 2F, G**); The TCGA prognosis analysis of TUBA1C gene was verified, and it was found that the prognosis of high expression of TUBA1C gene was poor (**Figure 2H**).

### Changes of TandNK before and after treatment

**Figure 3:**
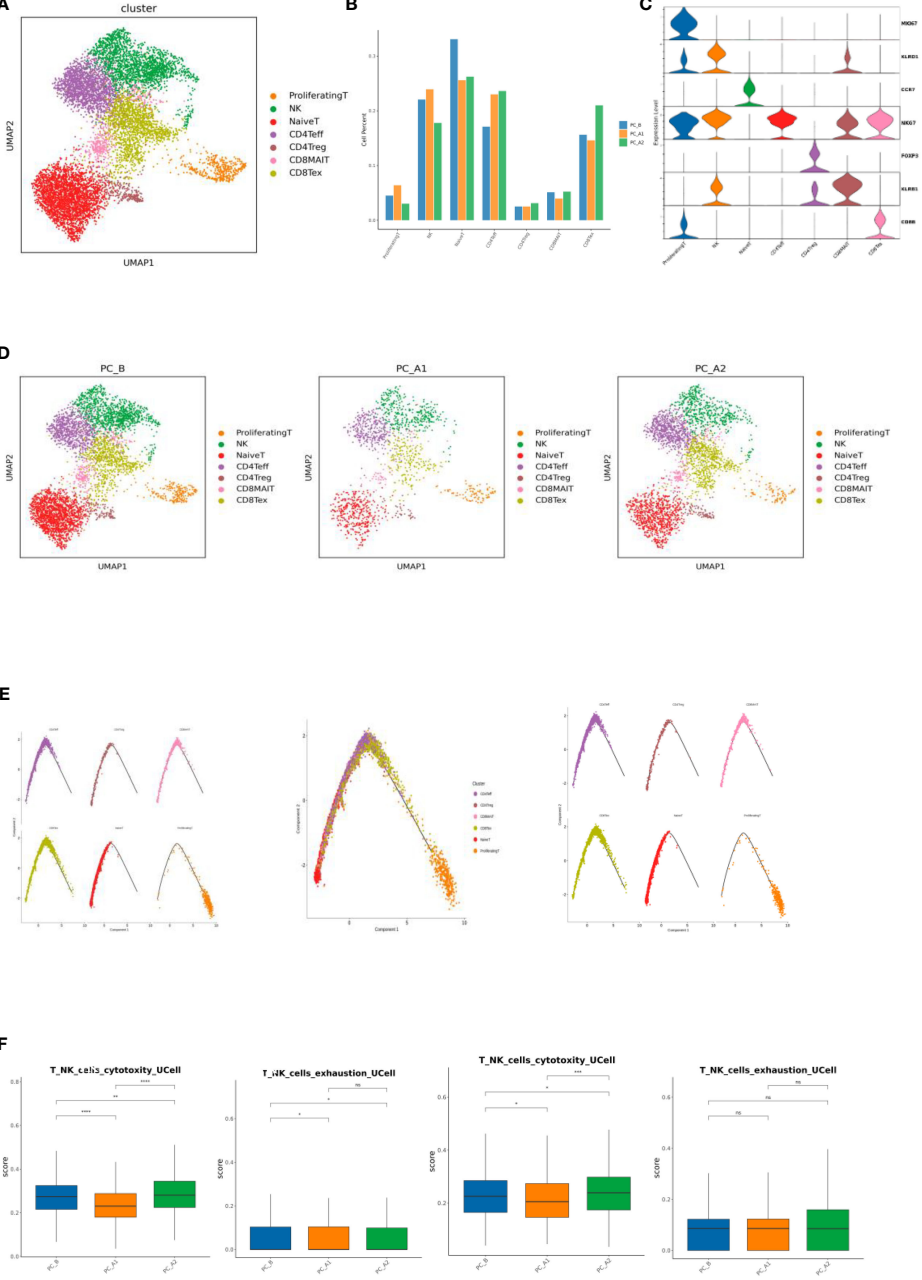
**(A)** T Cell Subtype vitality Reduction Map (UMAP). **(B)** T cell subtype proportion bar chart. **(C)** T cell subtype marker gene violin map. **(D)** Group display of T cell subpopulation type Reduction map (UMAP). **(E)** T Cell subpopulation locus Analysis (monocle2). **(F)** Immune cell signature base.

TandNK has 9152 cells in total, and 7 different subtypes have been subdivided, including Proliferating T, NK, NaiveT, CD4Teff, CD4Treg, CD8MAIT, and CD8Tex (**Figures 3A, C, D**). With the course of treatment, the proportion of CD4Treg and CD4Teff gradually increased, while the proportion of CD8Tex first decreased and then increased, and the proportion of NK first increased and then decreased (**Figure 3B**).

Differentiation and development of different T cells were analyzed by monocle2, it was found that T differentiation and development began in NaiveT cells, followed by effector T cells and depletion T cells (**Figure 3E**). Gene set scoring Gene set scoring was performed using the R package UCell v 1.1.0 (1). UCell scores are based on the Mann-Whitney U statistic by ranking query genes’ in order of their expression levels in individual cells. Because UCell is a rank-based scoring method, it is suitable to be used in large datasets containing multiple samples and batches (14), through Ucell analysis, it was found that NK and CD8Tex cytotoxicity scores decreased first and then increased with the course of treatment, while NK cell depletion scores decreased with the course of treatment, and CD8Tex cell depletion scores did not change significantly (**Figure 3F**).

### Changes of MPs before and after treatment-macrophages

**Figure 4:**
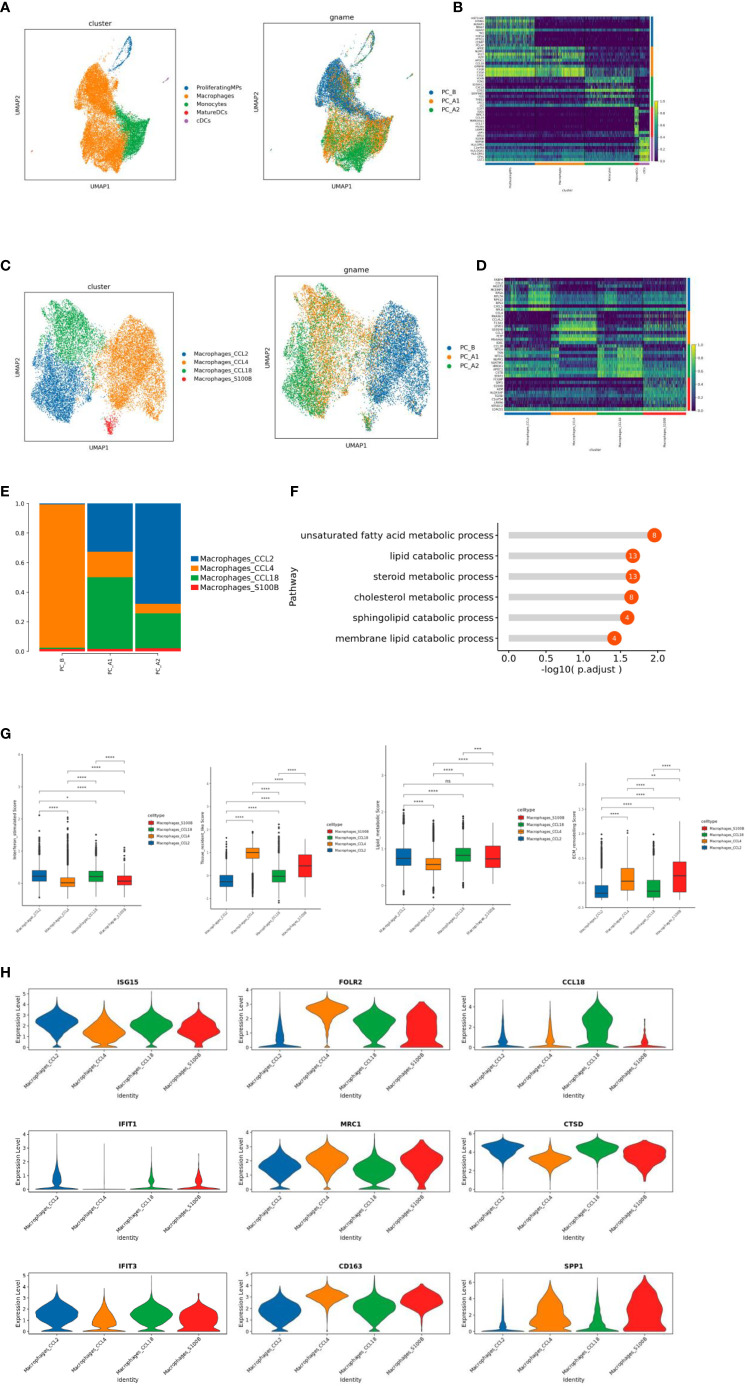
**(A)** ps subtype vitalization reduction map. **(B)** Heat map of Top10 differential genes in MPs subpopulation. **(C)** Macrophage subtype vitreogram (overall and group). **(D)** Heat map of Top10 differential genes of macrophage subtypes. **(E)** Histogram of the proportion of macrophage subtypes. **(F)** GO enrichment analysis of CCL18 subgroup. **(G)** Scores of characteristic gene sets of macrophage subtypes (g-1 to g-4 sequence: interferon-responsive macrophages, tissue-resident macrophages, lipid metabolizing macrophages, and extracellular matrix remodeling function macrophages). **(H)** Violin map of characteristic genes of different macrophage subpopulations.

There are 21447 cells in total, and 5 distinct subtypes are derived, including Proliferating MPs, Macrophages, Monocytes, MatureDCs, and cDCs (**Figures 4A, B**). Macrophages of a total of 16326 cells presented an unsupervised clustering result and were divided into 4 heterogeneous subpopulations (CCL2, CCL4, CCL18, S100B) (**Figures 4C–E**). GO pathway enrichment analysis showed that CCL18 subgroup up-regulated genes were enriched in lipid metabolism pathways (**Figure 4F**).

The CCL2 subgroup of highly expressed genes is related to IFN response and regulation (ISG15, IFIT1, IFIT3), which significantly increased the interferon response score (**Figures 4G-1, H**). CCL4 subgroup is a tissue-resident macrophage with high expression of FOLR2, MRC1 and CD163 genes (**Figure 4G-2, H**). CCL18 subgroup highly expressed CCL18 and CTSD genes, was a lipid metabolizing type of macrophage (corresponding to the results of functional enrichment analysis of the subgroup) (**Figure 4G-3, H**). S1008 subgroup highly expressed SPP1 gene, which significantly increased the score of extracellular matrix remodeling function (**Figure 4G-4, H**).

### Changes of MPs before and after treatment-monocytes

**Figure 5:**
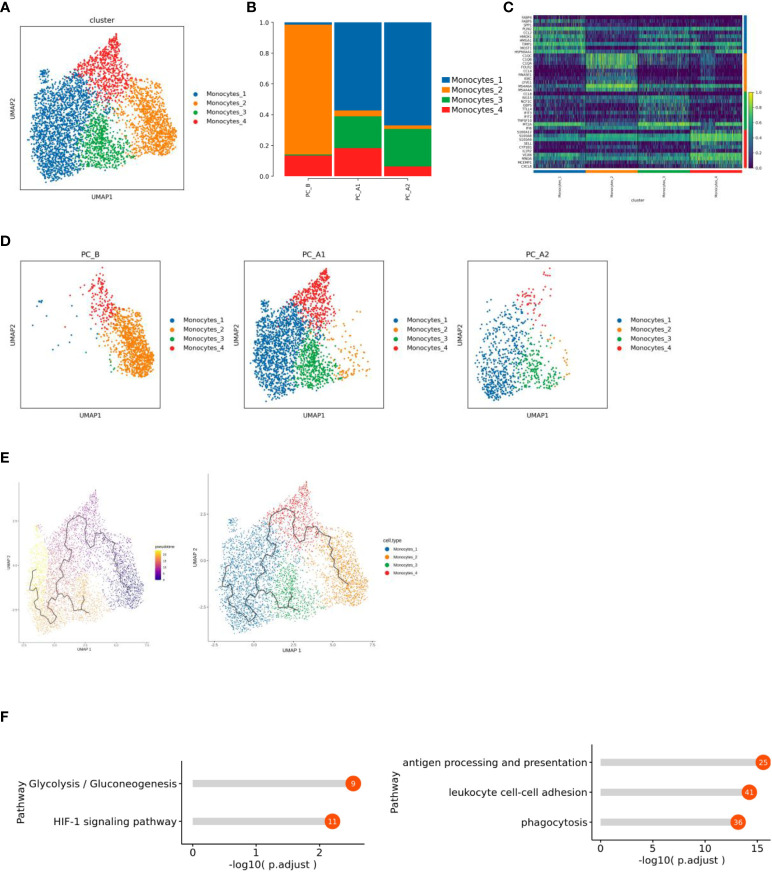
**(A)** Monocyte subtype vitreogram. **(B)** Monocyte subtype proportion histogram. **(C)** Heat map of Top10 differential genes of monocyte subtypes. **(D)** Monocyte subtype group dimension reduction map (display sequence: PC_B, PC_A1, PC_A2). **(E)** Quasi-time series analysis of monocyte subtypes. **(F)** Functional enrichment results of Monocytes_1 and 2 subgroups.

Monocytes consist of 4561 cells, which are subdivided and noted into four different subtypes, of which subgroup 2 is mainly sampled before the treatment, while subgroups 1 and 3 are mainly sampled after the treatment (**Figures 5A–D**). The monocle3 locus analysis of all monocyte subsets showed that, with the course of treatment, subgroup 2 at the starting point of development gradually differentiated into subgroup 4 in the middle state, and subgroup 3 and subgroup 1 were at the end of differentiation and development (**Figure 5E**).

Functional enrichment analysis of subgroup differential genes showed that subgroup 1 showed increased enrichment in HIF-1 signaling pathway and glycolysis/gluconeogenesis pathway (**Figure 5F**).

### Changes of neutrophils before and after treatment

**Figure 6:**
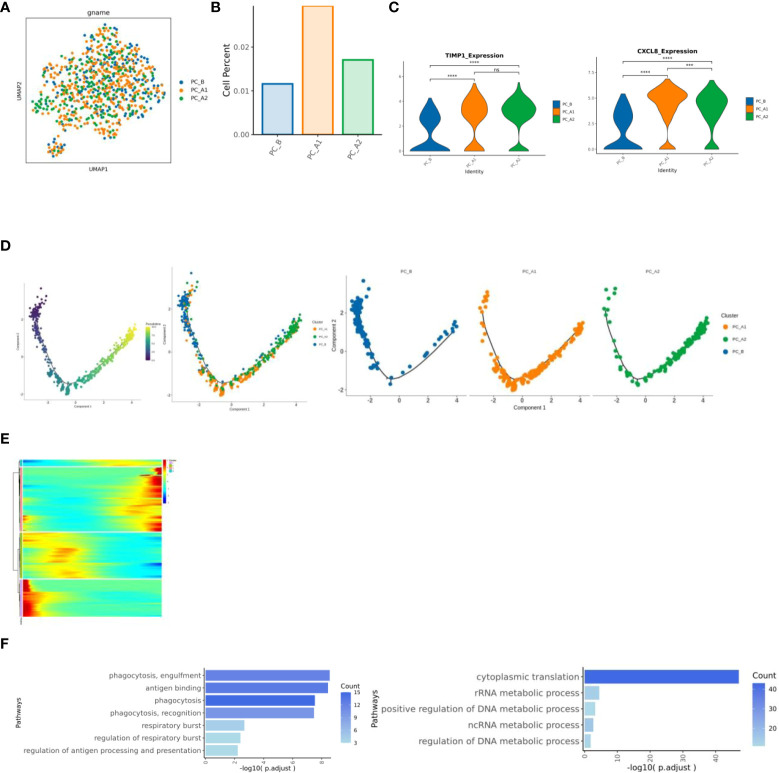
**(A)** neutrophil group reduction map. **(B)** Histogram of the proportion of neutrophil groups. **(C)** Results of expression differences between specified genomes (violin diagram). **(D)** Neutrophil locus analysis results (monocle2). **(E)** Heat map of dynamic change of gene expression with pseudo time change. **(F)** Results of cluster1 and cluster3 enrichment analysis based on pseudo time variation.

With the course of treatment, the proportion of Neutrophils showed a trend of first increasing and then decreasing (**Figures 6A, B**). Inter-group differential gene analysis showed that the expression of CXCL8 gene and TIMP1 gene increased significantly with the course of treatment (**Figure 6C**). Monocle2 locus analysis showed that PC_B was in the early stage of development, while PC_A1 and PC_A2 were in the late stage of development (**Figure 6D**). The functional enrichment analysis was carried out based on the cluster of dynamic change heat maps of Monocle2 locus, and the analysis found that: cluster1 in early development is enriched in phagocytosis, respiratory burst, and antigen presentation, cluster3 in late development are significantly up regulated in the metabolic pathway, involved in the occurrence and development of tumors, and showed a pro-tumor effect (**Figures 6E, F**).

### The influence of SSG on the expression of MHC-II in epithelial cells

**Figure 7:**
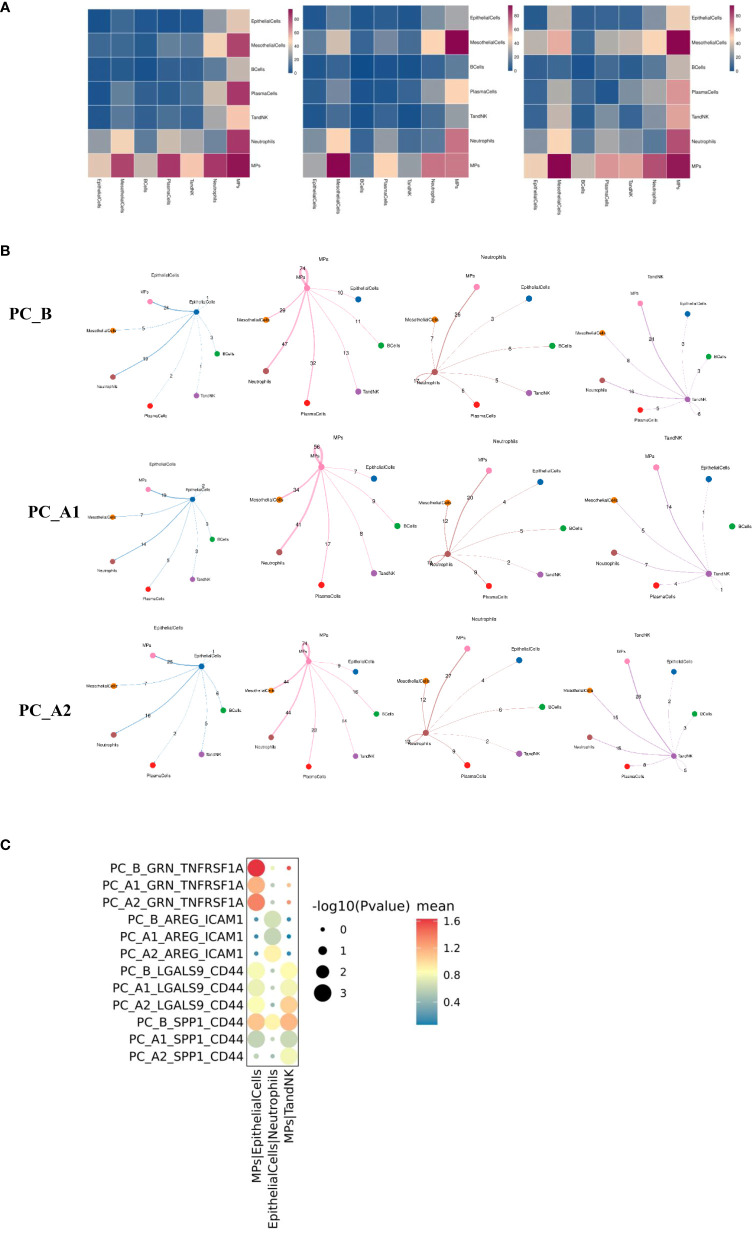
**(A)** Group display of all major types of cells interacting quantity heat map display (display sequence PC_B, PC_A1, PC_A2). **(B)** Shell diagrams showing interactions of epithelial cells, mononuclear phagocytes, neutrophils, TandNK and other cells in groups (Display sequence PC_B, PC_A1, PC_A2 from top to bottom). **(C)** The signal intensity of GRN_TNFRSF1A, AREG_ICAM1, LGALS9_CD44 and SPP1_CD44 between epithelial cells and mononuclear phagocytes changed with the course of treatment.

MHC-II_SIGNALING” gene set analysis and corresponding gene set expression, epithelial grouping MHC-II gene set scoring (UCell) and CD 73 and 74 for epithelial grouping MHC-II gene expression”. The trend of gene set scoring results and gene expression results is to decrease first and then increase (**Figure 7**) (15).

### Cell interactions (CellphoneDB) before and after treatment

The interactions of various types of cell clusters in the three groups of samples were analyzed using the CellPhoneDB database based on ligand-receptor interaction, and the results showed that the epithelial-mononuclear phagocytes, epithelial-neutrophils, and mononuclear phagocyte-TandNK interactions first decreased and then increased with the course of treatment (**Figure 7**).

Further analysis showed that the GRN_TNFRSF1A signal intensity between epithelial cells and mononuclear phagocytes decreased first and then increased with the course of treatment. The signal intensity of LGALS9_CD44 and SPP1_CD44 between mononuclear phagocytes and TandNK cells also decreased first and then increased with the course of treatment. It has been reported that macrophages can regulate T cell activity through various signaling pathways such as SPP1-CD44 and LGALS9-CD44.

### The influence of SSG on the expression of MHC-II in epithelial cells

**Figure 8:**
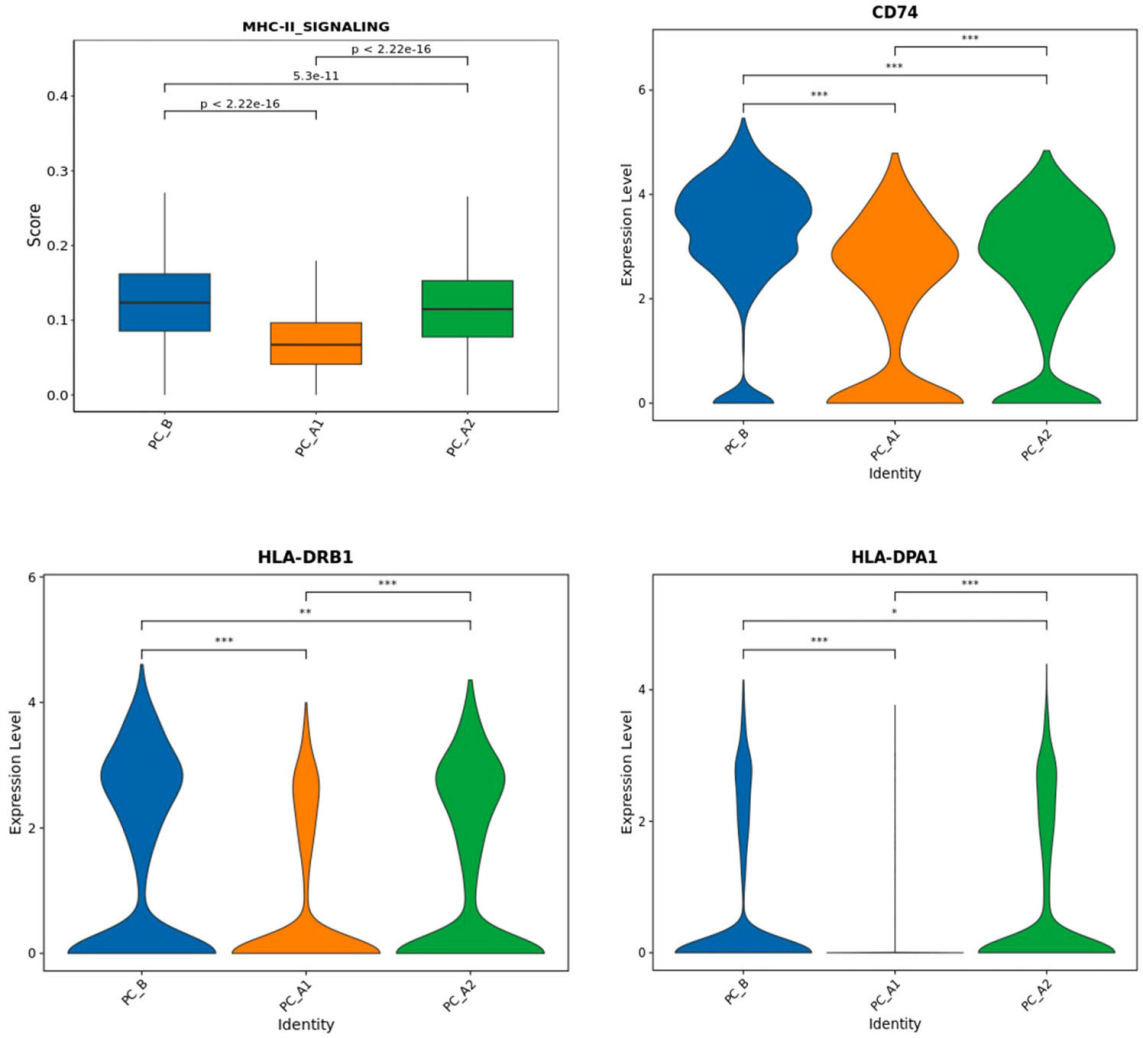
The expression of signature genes related to MHC-II pathway (CD74, HLA-DPA 1, HLA-DRB 1) decreased and then increased with the treatment process (Figure i), and Ucell analysis found that the epithelial MHC-II pathway score decreased and then increased with the treatment process.

MHC-II_SIGNALING” gene set analysis and corresponding gene set expression, epithelial grouping MHC-II gene set scoring (UCell) and CD 73 and 74 for epithelial grouping MHC-II gene expression”. The trend of gene set scoring results and gene expression results is to decrease first and then increase (**Figure 8**) (15).

## Discussion

Sodium stibogluconate (SSG) has been used for treating visceral leishmaniasis for approximately half a century. Pentostam a.k.a sodium stibogluconate is the pentavalent antimonial compound available in the United States (through the Centers for Disease Control). With the development of dosing regimens for the treatment of leishmaniasis, the daily dose and duration of treatment for antimony have been progressively increased to achieve a complete response to treatment (5, 6). SSG is a small molecule inhibitor of protein tyrosine phosphatases (PTPs), that cab be used in combination with IFN-alpha-2b (IFN-α) for inhibiting solid tumor cell line growth *in vitro*. A Phase I clinical trial with SSG plus IFN-αin advanced cancer patients to assess tolerance, maximum tolerated dose (MTD) and immune system effects were evaluated. The results showed that SSG in combination with IFN-α2b was well tolerated and augmented cellular immune parameters (16). PTPs is ubiquitously expressed in most cells and regulates cell proliferation as well as differentiation in the physiology of multicellular organisms. PTPs can regulate the intracellular signaling mechanism of immune cells via dephosphorylation of multiple targets, PTPs also affect disease through their role in innate and/or acquired immunity (17). SSG was evaluated for immune response of Leishmania donovani infected BALB/c mice and it showed that Only SSG NIV (non-ionic surfactant vesicular) treated animals gave a significant positive DTH response to an L. donovani parasite preparation (P < 0.05) (18, 19).

In this study, we used an scRNA-Seq for analysis of malignant epithelial cells and immune cells, including Epithelial Cells, Ecs, Fibroblasts, Mural Cells, Tcells, Bcells, TandNK Neutrophils, Mast Cells, MPs, and Platelets, as well as the dynamic changes in cell percentage and cell subtype heterogeneity before and after SSG was injected with single-drug into pleural effusion. It indicated the proportion of epithelial cells and TandNK cells first decreased and then increased, the proportion of neutrophils and mononuclear phagocytes first increased and then decreased (**Figure 1B**), and the proportion of plasma cells decreased following the course of SSG treatment. These results suggest that SSG therapy induces the remodeling of the pleural fluid microenvironment in patients and opens a new window for the next treatment.

Through cell proportion, CNV and ITH analysis, we found that pleuroepithelial cells of patients with different treatment processes showed obvious heterogeneity. Significant active regulators in the epithelial cell subtype transcription factor (TF) regulatory network were evaluated by pyscenic analysis, and cluster 2 was found to up-regulate the proto-oncogene REL transcription factor activity (20).

The proportion of CD4Treg and CD4Teff gradually increased, while the proportion of CD8Tex first decreased and then increased, and the proportion of NK first increased and then decreased; through Ucell analysis, it was found that NK and CD8Tex cytotoxicity scores decreased first and then increased with the course of treatment, while NK cell depletion scores decreased with the course of treatment, and CD8Tex cell depletion scores did not change significantly. Regulatory and run out of T cells mainly in the enrichment of T-cell development later, state shows that T cells from activation to suppress and exhausted, consistent with previous studies (21).

The study indicated that MPs included proliferating MPs, Macrophages, Monocytes, MatureDCs, and cDCs (**Figures 4A–E**), and analyzed by GO pathway enrichment, it has showed that CCL18 subgroup up-regulated genes were enriched in lipid metabolism pathways. Some studies have reported that lipid metabolism can regulate phagocytosis of macrophages and the production of inflammatory factors (22).

The CCL2 subgroup highly expressed genes related to IFN response and regulation (ISG15, IFIT1, IFIT3), which significantly increased the interferon response score (**Figures 4G-1, H**). Studies have shown that interferon-responsive macrophages have an immunosuppressive effect, which inhibits the immune response through tryptophan degradation and recruitment of immunosuppressive regulatory T cells (Tregs) (23). and the proportion increases gradually with the course of treatment.

CCL4 subgroup is a tissue-resident macrophage with high expression of FOLR2, MRC1 and CD163 genes (**Figures 4G-2, H**). Studies have shown that the density of FOLR2+ macrophages in breast cancer is positively correlated with better patient survival (18). Along with the treatment process of gradually reduce. S1008 subgroup highly expressed SPP1 gene, which significantly increased the score of extracellular matrix remodeling function (**Figures 4G-4, H**). Cell surface receptors interact with ECM components active ECM binding factors, which can receive cell adhesion and signal transduction, thus regulating cell proliferation, differentiation, migration, apoptosis and other processes (24), and the proportion gradually increased with the course of treatment.

The present study indicates that monocytes is one of MPs, which consist of 4561 cells subdivided and noted into four different subtypes, of which subgroup 2 is mainly sampled before the treatment, while subgroups 1 and 3 are mainly sampled after the treatment. Functional enrichment analysis of subgroup differential genes showed that subgroup 1 showed increased enrichment in HIF-1 signaling pathway and glycolysis/gluconeogenesis pathway. It has been reported that activated HIF-1 activated downstream genes that regulate important biological processes required for tumor survival and development (including glucose metabolism, cell proliferation, migration and angiogenesis) through transcription. 2 subgroups in antigen processing and rendering, enrichment of phagocytosis and intercellular adhesion (24, 25).

The proportion of neutrophils showed a trend of first increasing and then decreasing following the course of treatment (**Figures 6A, B**). Inter-group differential gene analysis showed that the expression of CXCL8 gene and TIMP1 gene increased significantly with the course of treatment. Some studies reported that higher CXCL8 expression level was associated with shorter overall survival and relapse-free survival (26), TIMP1 gene is associated with poor prognosis of pancreatic ductal adenocarcinoma (27).

Monocle2 locus analysis showed that PC_B was in the early stage of development, while PC_A1 and PC_A2 were in the late stage of development. The functional enrichment analysis was carried out based on the cluster of dynamic change heat maps of Monocle2 locus, and the analysis found that: cluster1 in early development is enriched in phagocytosis, respiratory burst, and antigen presentation. Studies have shown that in the early and middle stages of pancreatic ductal adenocarcinoma invasion, tumor-associated neutrophil TAN-1 is enriched in genes involved in phagocytosis, antigen presentation, and respiratory burst (28), cluster3 in late development is significantly up-regulated in the metabolic pathway, involved in the occurrence and development of tumors, and showed a pro-tumor effect (29).

SSG fusion into treatment of ascites and pleural effusion of pancreatic cancer has strength the interaction between the immune cells, NK and CD8Tex cytotoxicity scores decreased, then increased with the course of treatment, while NK cell depletion scores decreased with the course of treatment, and CD8Tex cell depletion scores did not change significantly. It has showed that MPs function, CCL18 subgroup up-regulated genes were enriched in lipid metabolism pathways. The CCL2 subgroup highly expressed genes related to IFN response and regulation significantly increased the interferon response score; CCL4 subgroup is a tissue-resident macrophage with high expression of FOLR2, MRC1 and CD163 genes; S1008 subgroup highly expressed SPP1 gene. It has shown that Monocytes function, the four different subtypes, of which subgroup 2 is mainly sampled before the treatment, while subgroups 1 and 3 are mainly sampled after the treatment. The influence of SSG on the expression of MHC-II in epithelial cells, as MHC-II is a marker for predicting immune therapy. It has shown that Neutrophils, a trend of first increasing and then decreasing following the course of treatment associated with the expression of CXCL8 gene and TIMP1 gene increased significantly with the course of treatment.

In summary, our current study shows that the interaction between TandNK cells, MPs cells, Monocytes, Neutrophil cells are induced during SSG fusion the treatment of ascites and pleural effusion, SSG promotes the expression of large numbers of genes and leads to upregulation of immune response. Our study provides evidence SSG can be used an immune adjuvant not only as a protein tyrosine phosphatase inhibitor in treatment of cancer patients, but also for recruiting the immune cells to the right place in fighting cancer cells in patients with ascites and pleural effusion with PD1 or PD-L1.

## Data availability statement

The original contributions presented in the study are publicly available. This data can be found here https://www.biosino.org/node/project/detail/OEP004994.

## Ethics statement

The studies involving humans were approved by Shandong Baofa Cancer Institute, Inc,. The studies were conducted in accordance with the local legislation and institutional requirements. Written informed consent for participation in this study was provided by the participants’ legal guardians/next of kin. Written informed consent was obtained from the individual(s) for the publication of any potentially identifiable images or data included in this article.

## Author contributions

BY: Conceptualization, Methodology, Writing – original draft. PJ: Formal Analysis, Investigation, Methodology, Resources, Writing – review & editing. FG: Data curation, Methodology, Project administration, Resources, Writing – original draft. PZ: Investigation, Project administration, Resources, Writing – review & editing. QZ: Methodology, Project administration, Resources, Software, Writing – review & editing. XZ: Investigation, Methodology, Project administration, Resources, Writing – review & editing.
